# Metabolic profiles of children aged 2–5 years born after frozen and fresh embryo transfer: A Chinese cohort study

**DOI:** 10.1371/journal.pmed.1004388

**Published:** 2024-06-06

**Authors:** Wei Zhou, Wanbing Feng, Jinli Chang, Jingmei Hu, Fuxia Li, Kuona Hu, Jiejing Jiao, Xinyi Xue, Ting Lan, Wenjing Wan, Zi-Jiang Chen, Linlin Cui

**Affiliations:** 1 State Key Laboratory of Reproductive Medicine and Offspring Health, Center for Reproductive Medicine, Institute of Women, Children and Reproductive Health, the Second Hospital, Shandong University, Jinan, Shandong, China; 2 National Research Center for Assisted Reproductive Technology and Reproductive Genetics, Shandong University, Jinan, Shandong, China; 3 Key laboratory of Reproductive Endocrinology (Shandong University), Ministry of Education, Jinan, Shandong, China; 4 Shandong Technology Innovation Center for Reproductive Health, Jinan, Shandong, China; 5 Shandong Provincial Clinical Research Center for Reproductive Health, Jinan, Shandong, China; 6 Shandong Key Laboratory of Reproductive Medicine, Shandong Provincial Hospital Affiliated to Shandong First Medical University, Jinan, Shandong, China; 7 Research Unit of Gametogenesis and Health of ART-Offspring, Chinese Academy of Medical Sciences (No.2021RU001), Jinan, Shandong, China; 8 Shanghai Key Laboratory for Assisted Reproduction and Reproductive Genetics, Shanghai, China; 9 Department of Reproductive Medicine, Ren Ji Hospital, Shanghai Jiao Tong University School of Medicine, Shanghai, China

## Abstract

**Background:**

Frozen embryo transfer (FET) has become a widely employed assisted reproductive technology technique. There have historically been concerns regarding the long-term metabolic safety of FET technology in offspring due to pregnancy-induced hypertension and large for gestational age, both of which are well-recognized factors for metabolic dysfunction of children. Therefore, we aimed to compare the metabolic profiles of children born after frozen versus fresh embryo transfer at 2 to 5 years of age.

**Methods and findings:**

This was a prospective cohort study. Using data from the “Assisted Reproductive Technology borned KIDs (ARTKID),” a birth cohort of offspring born from assisted reproductive technology at the Institute of Women, Children and Reproductive Health, Shandong University, China. We included 4,246 singletons born after FET (*n* = 2,181) and fresh embryo transfer (*n* = 2,065) enrolled between 2008 and 2019 and assessed the glucose and lipid variables until the age of 2 to 5 years. During a mean follow-up of 3.6 years, no significant differences were observed in fasting blood glucose, fasting insulin, Homeostatic Model Assessment of Insulin Resistance Index, total cholesterol, triglycerides, low-density lipoprotein-cholesterol, and high-density lipoprotein-cholesterol levels between offspring conceived by fresh and frozen embryo transfer in the crude model and adjusted model (adjusted for parental age, parental body mass index, parental education level, paternal smoking, parity, offspring age and sex). These results remained consistent across subgroup analyses considering offspring age, the stage of embryo transfer, and the mode of fertilization. Results from sensitivity analysis on children matched for age within the cohort remains the same. The main limitation of our study is the young age of the offspring.

**Conclusions:**

In this study, the impact of FET on glucose and lipid profiles during early childhood was comparable to fresh embryo transfer. Long-term studies are needed to evaluate the metabolic health of offspring born after FET.

## Abstract 2

Wei Zhou and co-workers explore the impact of fresh Versus frozen embryo transfer on the metabolic profiles of children in China.

## Author summary

**Why was this study done?:**

- Frozen embryo transfer (FET) is a commonly used assisted reproductive technology technique.
- Concerns have existed about the long-term metabolism of FET for offspring due to increased risk of well-recognized factors for metabolic dysfunction, including pregnancy-induced hypertension, macrosomia, large for gestational age, and elevated birth weight.
- Limited studies have reported on the metabolic profiles of children born after FET, leaving the potential long-term metabolic safety of FET unclear.

**What did the researchers do and find?:**

- Using data from the “Assisted Reproductive Technology borned KIDs (ARTKID),” a prospective birth cohort at a Chinese single reproductive center, 4,246 singletons of “ARTKID” cohort at 2 to 5 years of age born after either FET (*n* = 2,181) or fresh embryo transfer (*n* = 2,065) were included.
- The average duration of follow-up across the study was 3.6 years. Assessments included fasting blood glucose, insulin, total cholesterol, triglycerides, low- and high-density lipoprotein cholesterol.
- The study found no significant differences in various metabolic parameters between children born after frozen versus fresh embryo transfer, even after adjusting for potential confounders, including parental age, body mass index, and other factors [adjusted mean difference (95% CI): fasting blood glucose, −0.01(−0.03, 0.02); total cholesterol, 0(−0.05, 0.04)].

**What do these findings mean?:**

- FET does not have a significant adverse impact on glucose and lipid profiles during early childhood.
- These data provide valuable evidence on the safety of FET when counselling couples undergoing assisted reproductive technology treatment.
- The main limitations are the young age of the offspring, as they may not fully manifest comprehensive metabolic phenotypic changes at this stage, necessitating longer follow-up studies for a more thorough understanding.

## Introduction

As the most widely used treatment for infertility, over 10 million children worldwide were conceived through assisted reproductive technology (ART) by 2022 [1]. Previous studies on offspring born via ART during early childhood have indicated an increased risk of impaired metabolism, including higher fasting blood glucose (FBG), insulin, triglycerides (TG), and lower low-density lipoprotein-cholesterol (LDL-C) levels [2–4]. According to the Developmental Origins of Health and Disease (DOHaD) theory, exposures during early life can lead to metabolic alterations that manifest later in life [5]. Therefore, it is essential to identify the key factors related to ART techniques that transmit metabolic risks to the next generation.

Frozen embryo transfer (FET) has become a well-established and widely used ART technique [6,7]. The use of FET is increasing substantially due to the reduced risk of ovarian hyperstimulation syndrome (OHSS), multiple gestations, and higher singleton livebirth rate [8–10]. However, compared to Fresh-ET, FET has shown an increased risk of pregnancy-induced hypertension (PIH), macrosomia, large for gestational age (LGA), and elevated birth weight [6,11–13]. All of these conditions are well-recognized risk factors for metabolic dysfunction and cardiovascular disease later in life [14–17]. FET was associated with a 1.5-fold increased risk of obesity than Fresh-ET offspring at age 5 to 8 years [18]. And, decreased body mass index (BMI) in offspring was observed in 2 to 3 aged offspring conceived by Fresh-ET but not FET compared with those who were naturally conceived [19]. Nevertheless, limited studies have reported on the metabolic profiles of children conceived through FET, especially in comparison to fresh controls. A cross-sectional study found more favourable lipid profiles were evident in fresh but not frozen prepubertal offspring compared with the naturally conceived counterparts [20], while another cohort study observed no differences among offspring born after fresh, frozen ET, and naturally conceived [9]. More importantly, given the limited sample size of these observed studies, the long-term metabolic safety of offspring born after FET is largely unknown.

Therefore, we conducted a prospective cohort study to compare the glucose and lipid profiles of children born following FET and Fresh-ET during early childhood.

## Material and methods

### Study design and participants

We conducted a prospective birth cohort of offspring born via ART, named “Assisted Reproductive Technology borned KIDs (ARTKID)” cohort, at the Institute of Women, Children and Reproductive Health, Shandong University, China. All the participants had assigned the informed consents for the follow-up before ART treatment started and the pregnancy and neonatal outcomes were recorded according to the request of our government (S1 and S2 Files). In 2013, we completed a further cohort design for adolescents and got the additional approval of the ethics committees at the Reproductive Medical Center of Shandong University (S3 File). All the data of kids were collected prospectively thereafter and analyzed until 2021. The “ARTKID” cohort focus on growth and metabolic health of children. The follow-up was conducted in 3 stages: infancy, toddlerhood, and puberty, with each stage comprising 2 to 3 follow-up visits based on age. Offspring who did not undergo growth assessment measurements are considered lost to follow-up. We contacted mothers via telephone and invited them, along with their offspring, to participate in our face-to-face visits. During follow-up, measurements of height and weight were taken to assess growth and development, relevant questionnaires were completed, and blood samples were provided. These assessments were performed by either well-trained pediatricians or nurses. And regular quality control measures were implemented to ensure the accuracy of the information collected.

**Fig 1:**
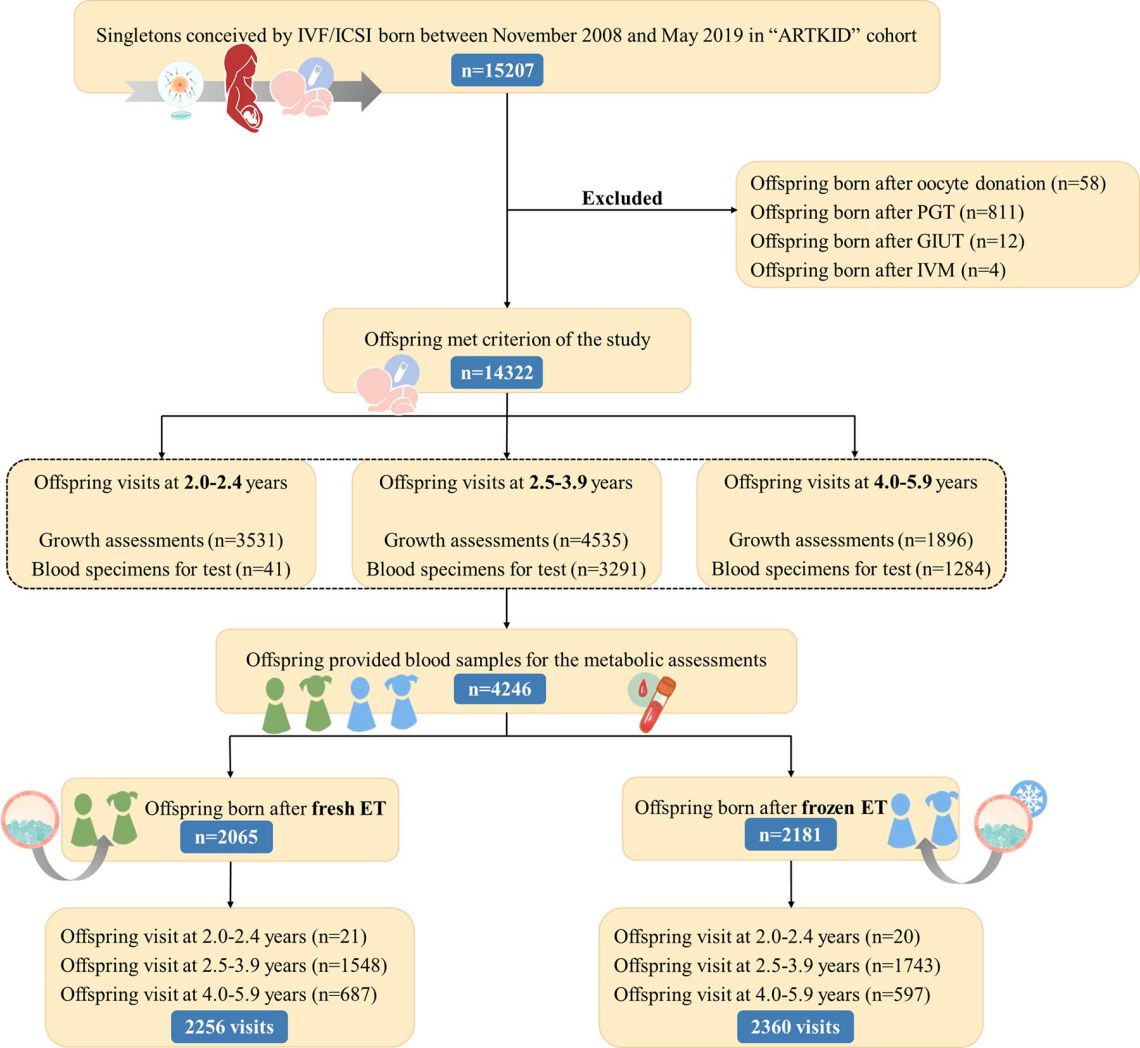
Flow chart of the study. Note: The follow-up stage during toddlerhood comprised 3 visits, encompassing ages 2.0–2.4, 2.5–3.9, and 4.0–5.9 years. In total, there were 9,962 visits from 8,765 offspring during the toddlerhood follow-up stage. All images/clip-art within the figure panels were hand-drawn by the authors. “ARTKID”, “Assisted Reproductive Technology borned KIDs”; ET, embryo transfer; GIUT, gamete intrauterine transfer; ICSI, intracytoplasmic sperm injection; IVF, in vitro fertilization; IVM, in vitro maturation; PGT, preimplantation genetic testing.

Offspring included in this study were born between November 2008 and May 2019 and were followed up until August 2021. The offspring included in this study were singletons conceived through either in vitro fertilization (IVF) or intracytoplasmic sperm injection (ICSI), and their ages ranged from 2 to 5 years on the follow-up day. Offspring conceived by oocyte donation, preimplantation genetic testing (PGT), gamete intrauterine transfer (GIUT), or in vitro maturation (IVM) were excluded from the study. In total, 14,322 singletons of the whole “ARTKID” cohort met the criterion. A total of 8,765 individuals completed growth assessments, and follow-up rate are 61.2% (8,765/14,322) until August 2021. Moreover, 2,181 children conceived by FET and 2,065 conceived by Fresh-ET provided blood samples for the assessment of glucose and lipid biochemical parameters and were included in the present analysis. All offspring born from FET used vitrification of embryos. In our longitudinal study, we implemented a 3 times follow-up design targeting specific age ranges: 2.0 to 2.4 years, 2.5 to 3.9 years, and 4.0 to 5.9 years. We required offspring to be followed up for at least once. Most offspring participated only once, and a few returned for follow-up assessments twice (183 children born from FET and 179 born from Fresh-ET) or 3 times (4 children born from FET). The mean length of follow-up across the cohort was 3.6 years, with a standard deviation of 0.8 years, and ranging from 2.0 to 5.9 years. And there were 97 sibling pairs (38 sibling pairs conceived by FET and 59 conceived by Fresh-ET) born to the same mothers included in our study. This study is reported as per the Strengthening the Reporting of Observational Studies in Epidemiology (STROBE) guideline (S1 Table). The flow chart of the study is showed in Fig 1.

Baseline characteristics included parental ages, BMI, diabetes status, education (college or higher, high school diploma or less), paternal smoking, parity (0 or more), stage of embryo transfer (cleavage or blastocyst), fertilization mode (IVF or ICSI), gestational diabetes mellitus (GDM), PIH, cesarean section, offspring sex (male or female), gestational age, birth weight, and age at visit. Parental characteristics were collected by standardized measurements and questionnaires administered during face-to-face interviews. ART treatment information was extracted by reviewing medical records. Parental ages at the time the child was born were calculated based on the date of parental birth and the offspring’s birth date. Information about offspring births was obtained from the offspring’s birth certificates. The birthweight values in standard deviation scores (SDS) were calculated based on the Chinese reference for sex- and gestational age-specific birthweight [21].

### Outcomes

The primary outcomes are FBG and LDL-C. FBG is crucial in diagnosing and managing diabetes [22]. LDL-C is the instigator of atherosclerotic plaques and main therapeutic target to prevent future cardiovascular events [23,24]. The secondary outcomes include insulin, The Homeostatic Model Assessment of Insulin Resistance Index using the HOMA2 Calculator (HOMA-IR2), TG, total cholesterol (TC), LDL-C, and high-density lipoprotein-cholesterol (HDL-C). Fasting blood samples were obtained in the morning and preserved at −80°C until subjected to analysis. FBG levels were assessed by the hexokinase method employing the Cobas c702 instrument (Roche Diagnostics, Germany). Fasting insulin levels were quantified in serum utilizing an electrochemiluminescence immunoassay conducted on the Cobas e601 instrument (Roche Diagnostics, Germany). Serum levels of TG, TC, LDL-C, and HDL-C were determined through a homogeneous assay employing the Cobas c702 instrument (Roche Diagnostics, Germany). The HOMA-IR2 score was computed using the HOMA calculator v2.2.3 (https://www2.dtu.ox.ac.uk/homacalculator/).

### Statistical analyses

Based on previous cohort and cross-sectional controlled data, the target mean differences were 0.09 mmol/L for FBG and 0.2 mmol/L for LDL-C, with standard deviations of 0.3 and 0.4, respectively [9,20]. The power was calculated using our sample size (*n* = 2,181 for FET and *n* = 2,065 for Fresh-ET) at a 2-sided alpha level of 0.05. The power was found to be nearly 100% for both FBG and LDL-C. Baseline characteristics between study offspring were compared using the linear regression for continuous variables and the logistic regression for categorical variables. The comparison of metabolic measurements between offspring conceived by fresh and frozen embryo transfer was used a linear mixed-effects regression model. The mixed-effects model allowed us to account for the potential confounding effects of 2 variations, including repeated measurements taken from the same child at different time points and siblings born to the same mothers. Potential confounders were defined based on priori literatures, including maternal age [25,26], paternal age [27], maternal BMI [28], paternal BMI [29], paternal smoking [30], parity [31], parental education [32], offspring age and sex [33,34]. Only less than 0.1% data were missing in the baseline variables and outcomes. Thus, the missing data were directly deleted in the final analysis.

Subgroup analyses were carried out according to offspring sex (male or female), the stages of embryo transfer (cleavage or blastocyst) and the mode of fertilization (IVF or ICSI). We conducted correlation analyses using Pearson’s correlation coefficient and found significant associations between FBG and insulin, as well as HOMA-IR2. Additionally, TC exhibited strong correlations with HDL and LDL levels (S2 Table). However, correlations with other indicators were not as pronounced. Therefore, Benjamin–Hochberg’s procedure was used to control false discovery rate (FDR) at a 5% level for multiple comparisons of FBG, TG, HDL, and LDL. To assess the robustness of study findings, we conducted a sensitivity analysis based on children matched for age within the study. This age matching was achieved through a one-to-one matching process employing nearest neighbour matching, with a calliper width set at 0.5 years.

The initial analysis conducted since September 2021 involved comparing metabolic variables between offspring born after fresh and frozen ET, as well as subgroup analyses based on the stages of embryo transfer (cleavage or blastocyst) and the mode of fertilization (IVF or ICSI), along with sensitivity analysis after matching for offspring age. However, considering the potential impact of offspring sex on metabolism, additional stratified analyses based on offspring sex (female or male) were conducted in February 2024. The statistical analyses were conducted using the R statistical software, version 4.2.3.

### Ethics approval

The study received approval from the ethics committees at the Reproductive Medical Center of Shandong University. All parents provided signed informed consent forms with the assent of the child.

## Results

Baseline characteristics of study offspring conceived by fresh and frozen embryo transfer are summarized in Table 1, encompassing parental, ART, pregnancy, and offspring characteristics. Maternal age and paternal age were significantly lower in offspring conceived by FET than those conceived by fresh embryos [maternal age: 30.9(28.4, 34.1) versus 31.3(28.4, 34.8); paternal age: 31.4(28.7, 34.9) versus 32.0(28.8, 35.6)]. Parental BMI, incidence of diabetes, educational level, paternal smoking status, and parity were comparable between offspring conceived by fresh and frozen embryo transfer. Offspring born after FET were more likely to undergo blastocyst ET and IVF than their fresh counterparts [blastocyst: 94% (2,051/2,181) versus 17.8% (368/2,065); IVF: 72.1% (1,572/2,181) versus 68.3% (1,410/2,065)]. Furthermore, mothers who received FET were more prone to developing PIH and undergoing cesarean sections compared to those who received fresh embryo transfer [PIH: 6.5% (141/2,181) versus 4.1% (84/2,065); cesarean sections: 74.9% (1,634/2,181) versus 72% (1,486/2,065)]. Birth weight was higher, and the age at the time of the visit was younger in the offspring conceived by frozen embryo transfer [birth weight SDS 0.55(−0.11, 1.31) versus 0.34(−0.30, 1.88); offspring age: 3.21(3.00, 4.01) versus 3.43(3.05, 4.16)]. There were no significant differences in the incidence of GDM, offspring sex distribution, or gestational age between offspring born after fresh and frozen embryo transfer.

**Table 1:** Characteristics of study population.

	Fresh embryo transfer (*n* = 2,065)	Frozen embryo transfer (*n* = 2,181)	MD or OR (95% CI)	*P* value
**Parental characteristics**				
Maternal age, years	31.3(28.4, 34.8)	30.9(28.4, 34.1)	−0.38(−0.63, −0.12)^#^	0.004
Paternal age, years	32.0(28.8, 35.6)	31.4(28.7, 34.9)	−0.42(−0.71, −0.13)^#^	0.004
Maternal BMI, kg/m^2^^**†**^	22.8(20.8, 25.2)	22.9(20.7, 25.4)	0(−0.21, 0.21)^#^	0.96
Paternal BMI, kg/m^2^^**†**^	25.5(23.0, 28.1)	25.6(22.9, 28.1)	0.01(−0.23, 0.26)^#^	0.91
Maternal diabetes, n (%)	7(0.3)	5(0.2)	0.68(0.21, 2.13)^§^	0.50
Paternal diabetes, n (%)	9(0.4)	9(0.4)	0.95(0.38, 2.39)^§^	0.91
Paternal smoking, n (%)	672(32.5)	704(32.3)	0.99(0.87, 1.12)^§^	0.85
Parity (0), n (%)	1,725(83.5)	1,797(82.4)	0.92(0.79, 1.08)^§^	0.32
Maternal education (college or higher), n (%)	662(32.1)	706(32.4)	1.01(0.89, 1.15)^§^	0.83
Paternal education (college or higher), n (%)	773(37.4)	790(36.2)	0.95(0.84, 1.08)^§^	0.41
**ART characteristics**				
Stage of embryo transfer			72.75(58.97, 89.76)^§^	<0.001
Cleavage, n (%)	1,697(82.2)	130(6.0)		
Blastocyst, n (%)	368(17.8)	2,051(94.0)		
Fertilization mode			0.83(0.73, 0.95)^§^	0.01
IVF, n (%)	1,410(68.3)	1,572(72.1)		
ICSI, n (%)	655(31.7)	609(27.9)		
**Pregnancy and offspring characteristics**				
GDM, n (%)	147(7.1)	176(8.1)	1.15(0.91, 1.44)^§^	0.24
PIH, n (%)	84(4.1)	141(6.5)	1.63(1.24, 2.15)^§^	0.001
Offspring sex (male), n (%)	1,080(52.3)	1,190(54.6)	1.10(0.97, 1.24)^§^	0.14
Cesarean section, n (%)	1,486(72.0)	1,634(74.9)	1.16(1.02, 1.33)^§^	0.03
Gestational age, weeks	39.1(38.4, 40.0)	39.1(38.4, 40.0)	0.01(−0.09, 0.11)^#^	0.81
Birth weight, SDS	0.34(−0.30, 1.88)	0.55(−0.11, 1.31)	0.14(0.07, 0.21)^#^	<0.001
Offspring age at visit, years	3.43(3.05, 4.16)	3.21(3.00, 4.01)	−0.13(−0.18, −0.09)^#^	<0.001

Data were presented as interquartile range (25th percentile, 75th percentile) or n (%).

MDs or OR were obtained using a regression model.

^#^For continuous variables, the regression results are presented as MD (95% CI).

^§^For categorical variables, the regression results are presented as OR (95% CI).

^**†**^Five data were missing in the maternal BMI, and 2 data were missing in the paternal BMI.

ART, assisted reproductive technology; BMI, body mass index; GDM, gestational diabetes mellitus; ICSI, intracytoplasmic sperm injection; IVF, in vitro fertilization; MD, mean difference; OR, odds ratio; PIH, pregnancy-induced hypertension syndrome; SDS, standard deviation scores.

We compared baseline characteristics between the included and not included offspring in the whole “ARTKID” cohort (S3 Table). Notably, our sample size is relatively large. Despite differences observed in parental age, paternal BMI, parity, embryo transfer stage, fertilization mode, and cesarean section rates, the actual difference were relatively small. For instance, the maternal age difference between those included and not included was merely 0.36 years in offspring conceived by fresh embryo transfer. The biochemical profile of offspring conceived by FET, compared with the fresh counterparts, is presented in Tables 2 and S4. No significant difference was observed in FBG, insulin, HOMA-IR2, TC, TG, and LDL-C levels between offspring conceived by fresh and frozen embryo transfer in crude and adjusted model. However, offspring born after FET showed a significantly increased HDL-C levels when compared to those born after Fresh-ET [mean difference (MD) (95% CI): crude model, 0.02(0.0005, 0.04); adjusted model, 0.02(0.002, 0.04)]. It is important to note that the significance of this difference was attenuated after the FDR correction (FDR q value: crude model, 0.16; adjusted model, 0.12).

**Table 2:** Differences in metabolic variables between offspring conceived by fresh versus frozen embryo transfer.

	Fresh embryo transfer (*n* = 2,065) (2,256 visits)	Frozen embryo transfer (*n* = 2,181) (2,360 visits)	Crude model MD (95% CI)	*P* value	Adjusted model MD (95% CI)	*P* value
FBG^**†**^, mmol/l	4.89 ± 0.44	4.88 ± 0.42	−0.02(−0.04, 0.01)	0.23	−0.01(−0.03, 0.02)	0.58
Insulin^**†**^, mIU/l	4.24 ± 3.06	4.16 ± 2.89	−0.08(−0.25, 0.09)	0.36	0.03(−0.14, 0.21)	0.70
HOMA-IR2^**†**^	0.56 ± 0.40	0.54 ± 0.38	−0.01(−0.03, 0.01)	0.36	0.004(−0.02, 0.03)	0.72
TC^**†**^, mmol/l	4.07 ± 0.73	4.07 ± 0.72	−0.01(−0.05, 0.04)	0.75	−0.004(−0.05, 0.04)	0.87
TG^**†**^, mmol/l	0.74 ± 0.29	0.73 ± 0.30	−0.01(−0.02, 0.01)	0.53	−0.003(−0.02, 0.01)	0.73
LDL-C^**†**^, mmol/l	2.45 ± 0.61	2.43 ± 0.60	−0.02(−0.06, 0.01)	0.21	−0.02(−0.06, 0.02)	0.26
HDL-C^**†**^, mmol/l	1.39 ± 0.29	1.41 ± 0.30	0.02(0.0005, 0.04)	0.04	0.02(0.002, 0.04)	0.03

Data were presented as mean ± SD.

MDs were obtained using a linear mixed-effects regression model.

Adjusted model: adjusted for maternal age, paternal age, maternal BMI, paternal BMI, maternal education, paternal education, paternal smoking, parity, offspring age and sex.

^**†**^Twenty-three data were missing in the FBG, 8 data were missing in the insulin, 30 data were missing in the HOMA-IR2, and 21 data were missing in the lipid metabolic variables.

FBG, fasting blood glucose; HDL-C, high-density lipoprotein cholesterol; HOMA-IR2, homeostatic model assessment for insulin resistance using the HOMA2 Calculator; LDL-C, low-density lipoprotein cholesterol; MD, mean difference; TC, total cholesterol; TG, triacylglycerol.

We stratified the offspring into 2 subgroups based on sex (female and male) (Tables 3 and S5). Within both subgroups, we found no significant differences in metabolic biochemical parameters between the study offspring even after adjusting parental characteristics and offspring age. Next, we stratified the population into 2 subgroups based on embryo transfer stage (cleavage and blastocyst) (Tables 4 and S6). Within both subgroups, we also found no significant differences in metabolic biochemical parameters between the study offspring. In the blastocyst ET subgroup, offspring born after FET had significantly higher TG levels than those born after Fresh-ET. However, this difference disappeared after FDR adjustment (FDR q value: adjusted model, 0.08). Furthermore, we grouped the children by fertilization mode (IVF and ICSI) (Tables 5 and S7). Similar to our previous analysis, there were no statistically significant interactions between fertilization mode and study offspring. In both IVF and ICSI subgroups, no significant differences were observed in any metabolic biochemical parameters between offspring conceived by fresh and frozen embryo transfer.

**Table 3:** Differences in metabolic variables between offspring conceived by fresh versus frozen embryo transfer stratified by offspring sex.

	Female offspring						Male offspring					
	**Fresh embryo transfer** **(*n* = 985)** **(1,067 visits)**	**Frozen embryo transfer** **(*n* = 991)** **(1,073 visits)**	**Crude model MD** **(95% CI)**	***P* value**	**Adjusted model MD** **(95% CI)**	***P* value**	**Fresh embryo transfer** **(*n* = 1,080)** **(1,189 visits)**	**Frozen embryo transfer** **(*n* = 1,190)** **(1,287 visits)**	**Crude model MD** **(95% CI)**	***P* value**	**Adjusted model MD** **(95% CI)**	***P* value**
FBG^**†**^, mmol/l	4.85 ± 0.43	4.83 ± 0.40	−0.02(−0.06, 0.02)	0.27	−0.01(−0.05, 0.03)	0.54	4.93 ± 0.46	4.91 ± 0.43	−0.01(−0.05, 0.02)	0.42	−0.003(−0.04, 0.03)	0.86
Insulin^**†**^, mIU/l	4.45 ± 3.14	4.42 ± 2.91	−0.04(−0.29, 0.22)	0.77	0.06(−0.19, 0.31)	0.63	4.06 ± 2.97	3.96 ± 2.86	−0.10(−0.34, 0.14)	0.40	0.01(−0.22, 0.24)	0.93
HOMA-IR2^**†**^	0.53 ± 0.39	0.52 ± 0.38	−0.005(−0.04, 0.03)	0.78	0.01(−0.03, 0.04)	0.64	0.58 ± 0.42	0.58 ± 0.38	−0.01(−0.05, 0.02)	0.38	0(−0.03, 0.03)	0.95
TC^**†**^, mmol/l	4.11 ± 0.74	4.10 ± 0.73	−0.02(−0.08, 0.04)	0.51	−0.01(−0.08, 0.05)	0.64	4.03 ± 0.72	4.04 ± 0.70	0.01(−0.05, 0.07)	0.79	0.01(−0.05, 0.07)	0.83
TG^**†**^, mmol/l	0.76 ± 0.29	0.75 ± 0.31	−0.01(−0.03, 0.02)	0.62	−0.01(−0.03, 0.02)	0.66	0.71 ± 0.28	0.71 ± 0.29	0(−0.03, 0.02)	0.80	0(−0.02, 0.02)	0.95
LDL-C^**†**^, mmol/l	2.51 ± 0.62	2.48 ± 0.62	−0.04(−0.09, 0.02)	0.19	−0.03(−0.09, 0.02)	0.23	2.40 ± 0.60	2.39 ± 0.60	−0.01(−0.06, 0.04)	0.74	−0.01(−0.06, 0.04)	0.66
HDL-C^**†**^, mmol/l	1.36 ± 0.29	1.38 ± 0.30	0.02(−0.01, 0.04)	0.23	0.02(−0.01, 0.04)	0.14	1.42 ± 0.29	1.43 ± 0.30	0.02(−0.01, 0.04)	0.14	0.02(−0.003, 0.04)	0.09

Data were presented as mean ± SD.

MDs were obtained using a linear mixed-effects regression model.

Adjusted model: adjusted for maternal age, paternal age, maternal BMI, paternal BMI, maternal education, paternal education, paternal smoking, parity, and offspring age.

^**†**^Twenty-three data were missing in the FBG, 8 data were missing in the insulin, 30 data were missing in the HOMA-IR2, and 21 data were missing in the lipid metabolic variables.

FBG, fasting blood glucose; HDL-C, high-density lipoprotein cholesterol; HOMA-IR2, homeostatic model assessment for insulin resistance using the HOMA2 Calculator; LDL-C, low-density lipoprotein cholesterol; MD, mean difference; TC, total cholesterol; TG, triacylglycerol.

**Table 4:** Differences in metabolic variables between offspring conceived by fresh versus frozen embryo transfer stratified by stage of embryo transfer.

	Cleavage						Blastocyst					
	**Fresh embryo transfer** **(*n* = 1,697)** **(1,848 visits)**	**Frozen embryo transfer** **(*n* = 130)** **(137 visits)**	**Crude model MD** **(95% CI)**	***P* value**	**Adjusted model MD** **(95% CI)**	***P* value**	**Fresh embryo transfer** **(*n* = 368)** **(408 visits)**	**Frozen embryo transfer** **(*n* = 2,051)** **(2,223 visits)**	**Crude model MD** **(95% CI)**	***P* value**	**Adjusted model MD** **(95% CI)**	***P* value**
FBG^**†**^, mmol/l	4.90 ± 0.45	4.89 ± 0.36	0(−0.08, 0.07)	0.90	−0.02(−0.09, 0.06)	0.68	4.85 ± 0.42	4.88 ± 0.42	0.03(−0.02, 0.07)	0.27	0.03(−0.02, 0.07)	0.24
Insulin^**†**^, mIU/l	4.31 ± 3.09	4.68 ± 2.66	0.37(−0.15, 0.90)	0.16	0.25(−0.27, 0.77)	0.35	3.93 ± 2.91	4.13 ± 2.90	0.22(−0.10, 0.54)	0.18	0.18(−0.13, 0.50)	0.25
HOMA-IR2^**†**^	0.56 ± 0.41	0.61 ± 0.35	0.04(−0.02, 0.11)	0.21	0.03(−0.04, 0.10)	0.40	0.52 ± 0.38	0.54 ± 0.38	0.03(−0.01, 0.07)	0.19	0.02(−0.02, 0.07)	0.26
TC^**†**^, mmol/l	4.08 ± 0.73	3.98 ± 0.69	−0.09(−0.22, 0.04)	0.15	−0.09(−0.22, 0.04)	0.18	4.03 ± 0.74	4.07 ± 0.72	0.03(−0.05, 0.11)	0.48	0.02(−0.05, 0.10)	0.55
TG^**†**^, mmol/l	0.75 ± 0.29	0.78 ± 0.28	0.04(−0.01, 0.09)	0.14	0.03(−0.02, 0.09)	0.20	0.68 ± 0.27	0.73 ± 0.30	0.05(0.01, 0.08)	0.004	0.04(0.01, 0.08)	0.01
LDL-C^**†**^, mmol/l	2.46 ± 0.61	2.42 ± 0.58	−0.04(−0.15, 0.07)	0.45	−0.04(−0.15, 0.07)	0.49	2.40 ± 0.63	2.43 ± 0.60	0.02(−0.05, 0.08)	0.62	0.01(−0.06, 0.08)	0.73
HDL-C^**†**^, mmol/l	1.38 ± 0.29	1.36 ± 0.29	−0.03(−0.08, 0.03)	0.33	−0.02(−0.08, 0.03)	0.35	1.43 ± 0.28	1.41 ± 0.30	−0.02(−0.05, 0.01)	0.29	−0.01(−0.05, 0.02)	0.37

Data were presented as mean ± SD.

MDs were obtained using a linear mixed-effects regression model.

Adjusted model: adjusted for maternal age, paternal age, maternal BMI, paternal BMI, maternal education, paternal education, paternal smoking, parity, offspring age and sex.

^**†**^Twenty-three data were missing in the FBG, 8 data were missing in the insulin, 30 data were missing in the HOMA-IR2, and 21 data were missing in the lipid metabolic variables.

FBG, fasting blood glucose; HDL-C, high-density lipoprotein cholesterol; HOMA-IR2, homeostatic model assessment for insulin resistance using the HOMA2 Calculator; LDL-C, low-density lipoprotein cholesterol; MD, mean difference; TC, total cholesterol; TG, triacylglycerol.

**Table 5:** Differences in metabolic variables between offspring conceived by fresh versus frozen embryo transfer stratified by fertilization mode.

	IVF						ICSI					
	**Fresh embryo transfer** **(*n* = 1,410)** **(1,538 visits)**	**Frozen embryo transfer** **(*n* = 1,572)** **(1,705 visits)**	**Crude model MD** **(95% CI)**	***P* value**	**Adjusted model MD** **(95% CI)**	***P* value**	**Fresh embryo transfer (*n* = 655)** **(718 visits)**	**Frozen embryo transfer** **(*n* = 609)** **(655 visits)**	**Crude model MD** **(95% CI)**	***P* value**	**Adjusted model MD** **(95% CI)**	***P* value**
FBG^**†**^, mmol/l	4.89 ± 0.46	4.88 ± 0.42	−0.02(−0.05, 0.02)	0.33	0(−0.03, 0.03)	0.82	4.89 ± 0.42	4.88 ± 0.42	−0.02(−0.06, 0.03)	0.49	−0.01(−0.06, 0.03)	0.53
Insulin^**†**^, mIU/l	4.24 ± 3.05	4.15 ± 2.74	−0.09(−0.30, 0.12)	0.40	0.04(−0.16, 0.25)	0.68	4.26 ± 3.08	4.21 ± 3.26	−0.06(−0.37, 0.26)	0.73	0.02(−0.29, 0.33)	0.90
HOMA-IR2^**†**^	0.55 ± 0.40	0.54 ± 0.36	−0.01(−0.04, 0.02)	0.39	0.01(−0.02, 0.03)	0.71	0.56 ± 0.40	0.55 ± 0.43	−0.01(−0.05, 0.03)	0.75	0(−0.04, 0.04)	0.90
TC^**†**^, mmol/l	4.07 ± 0.73	4.08 ± 0.72	0(−0.05, 0.05)	0.96	0(−0.05, 0.05)	0.99	4.07 ± 0.73	4.05 ± 0.70	−0.03(−0.11, 0.05)	0.48	−0.01(−0.09, 0.06)	0.72
TG^**†**^, mmol/l	0.74 ± 0.29	0.73 ± 0.29	−0.01(−0.03, 0.01)	0.36	−0.01(−0.03, 0.01)	0.49	0.73 ± 0.28	0.74 ± 0.33	0.005(−0.03, 0.04)	0.77	0.01(−0.02, 0.04)	0.66
LDL-C^**†**^, mmol/l	2.46 ± 0.62	2.44 ± 0.61	−0.01(−0.06, 0.03)	0.51	−0.02(−0.06, 0.03)	0.44	2.45 ± 0.60	2.40 ± 0.58	−0.05(−0.11, 0.02)	0.18	−0.03(−0.10, 0.03)	0.33
HDL-C^**†**^, mmol/l	1.39 ± 0.29	1.41 ± 0.30	0.02(−0.003, 0.04)	0.10	0.02(−0.0001, 0.04)	0.06	1.39 ± 0.29	1.41 ± 0.29	0.02(−0.01, 0.05)	0.26	0.02(−0.01, 0.05)	0.22

Data were presented as mean ± SD.

MDs were obtained using a linear mixed-effects regression model.

Adjusted model: adjusted for maternal age, paternal age, maternal BMI, paternal BMI, maternal education, paternal education, paternal smoking, parity, offspring age and sex.

^†^Twenty-three data were missing in the FBG, 8 data were missing in the insulin, 30 data were missing in the HOMA-IR2, and 21 data were missing in the lipid metabolic variables.

FBG, fasting blood glucose; HDL-C, high-density lipoprotein cholesterol; HOMA-IR2, homeostatic model assessment for insulin resistance using the HOMA2 Calculator; LDL-C, low-density lipoprotein cholesterol; MD, mean difference; TC, total cholesterol; TG, triacylglycerol.

Considering the offspring age in offspring conceived by FET was notably younger compared to the fresh counterparts (3.52 ± 0.74 versus 3.66 ± 0.74, *p* < 0.001), we conducted a sensitivity analysis on children matched for age within the cohort. After age-matching, 3,912 offspring were included, resulting in similar ages for both the offspring conceived by fresh and frozen embryo transfer (3.58 ± 0.76 versus 3.59 ± 0.72, *p* = 0.45). Consistent with our prior findings, we found no statistically significant differences in metabolic biochemical parameters between offspring born after fresh and frozen embryo transfer (S8 and S9 Tables).

## Discussion

The long-term safety of FET technology has historically been a concern because of adverse perinatal outcomes. Fortunately, our findings found no significant effect of frozen versus fresh embryo transfer on glucose and lipid biochemical parameters during early childhood.

Previous research has yielded inconsistent findings regarding the effect of FET on offspring metabolism. A Swedish study explored the risk of type 1 diabetes in singletons born after ART and found that offspring conceived by FET showed an increased risk of type 1 diabetes than those after Fresh-ET before 18 years in subgroup analysis (adjusted hazard ratio 1.52; 95% CI, 1.08 to 2.14) [35]. A New Zealand study reported that 115 children aged 3.5 to 11.0 years conceived through FET showed unfavourable lipid metabolism compared to those conceived through Fresh-ET, including higher TG and lower HDL-C [20]. Moreover, a rodent study has also observed that male offspring conceived by FET exhibit insulin resistance compared to their fresh counterparts [36].

In contrast, our study found no notable differences in glucose and lipid levels between 4,246 singletons aged 2 to 5 years born from frozen or fresh ET. The finding remained the same after adjusting for factors like parental, perinatal, and offspring characteristics. Several studies supported our research finding. A Finnish study observed that the endocrine, nutritional, and metabolic diseases in singletons born after FET do not differ from those born after Fresh-ET, with a mean follow-up time of 18 to 20 years [37]. A Danish cohort study found no statistically significant glucose and lipid profile differences among 150 singletons aged 8 to 9 years conceived after FET, Fresh-ET, and naturally conceived children [9]. These contradictory findings may be attributed to differences in sample size, offspring ethnicity, age, and potential confounding factors.

A higher proportion of blastocysts is transferred as frozen-thawed embryos [38]. Two previous meta-analyses have reported a significant higher risk of LGA in offspring born after blastocyst-stage embryo transfer compared with cleavage-stage transfer in frozen cycles, but not in fresh cycles [38,39]. Considering the potential different impact of embryo stage on the metabolic profiles of offspring, we conducted a subgroup analysis in children born after different stage. However, no significant differences in the metabolic profiles between frozen and Fresh-ET in subgroup analysis. And no interaction effects were found between these procedures and study offspring.

A study conducted by Green and colleagues identified adverse lipid metabolism among children conceived through FET than those conceived via Fresh-ET at the cleavage stage, including higher TG levels and lower HDL-C levels [20]. The study focused on children born between 1993 and 2005. Embryo cryopreservation, storage, and thawing techniques have significantly evolved in recent years, which may have contributed to the improvement of the metabolic health of frozen embryo offspring. Sample size and ethnic differences may also explain the inconsistent results. It is worth noting that there is a significantly higher proportion of blastocyst transfers among children born after FET and Fresh-ET [the proportion of blastocyst transfers: 94% (2,051/2,181) versus 17.8% (368/2,065)]. This disparity limited the statistical power and robustness of the findings within the embryo transfer stage subgroup. Consequently, these findings necessitate larger sample cohorts to be confirmed.

The gamete manipulation in ICSI may lead to epigenetic and imprinting alterations in the embryo [40]. A retrospective cohort study showed that birth weight of offspring born after ICSI was lower compared to those born after IVF [41]. Another recent study conducted by Catford and colleagues reported a significantly lower fasting glucose in 121 ICSI-conceived singletons compared with 74 IVF-conceived controls aged 18 to 24 years [42]. In our study, the proportion of ICSI was lower in offspring conceived by FET. Therefore, we further conducted subgroup analysis on the fertilization model to mitigate the potential impact of this confounding factor. These results remained consistent with the overall comparison of study offspring, further substantiating the robustness of the principal conclusion.

Our findings suggested there were no significant differences in glucose and lipid metabolism between fresh and frozen embryo offspring. However, previous studies have indicated that compared to natural conceived offspring, children conceived through FET or Fresh-ET often exhibit metabolic differences. Prior research highlights differences in metabolic markers, such as higher levels of FBG and Apolipoprotein E (APOE), as well as lower levels of HDL-C in children conceived through FET [2,20,43]. Children conceived through Fresh-ET also tend to have less favourable glucose and cardiovascular metabolic profiles than naturally conceived children [2]. Our study provides valuable insights into the strategy of embryo transfer for infertile couples undergoing IVF/ICSI. But the long-term metabolic health of offspring conceived through FET remains a concern, and continuous monitoring of their long-term metabolic health is necessary.

In comparison to prior related studies, our study exhibits several strengths. Firstly, the substantial sample size of 4,246 children enhances the robustness of our conclusions by providing a higher level of statistical power. Additionally, the large sample size enables us to conduct meaningful subgroup analyses. This provides more comprehensive information for both infertility couples and clinicians when discussing the pros and cons of various transfer and fertilized techniques. Secondly, our study focused on children aged 2 to 5 years, ensuring that they were not affected by minipuberty (1 to 6 months) or undergoing pubertal development. Finally, the data were highly homogeneous due to the use of single laboratory measurements for all participants and tests, which helped reduce confounding effects to some extent.

However, the study has certain limitations. Firstly, our study was based on an ART birth cohort and we did not include naturally conceived offspring in this cohort. Thus, it was not possible for us to include offspring conceived naturally. Our study focused on the strategy of embryo transfer for couples undergoing ART treatment. Secondly, the substantial disparity was observed in the stages of embryo transfer between offspring born after fresh and frozen embryo transfer. In our study, Fresh-ET primarily at the cleavage stage and FET at the blastocyst stage, which is consistent with other studies [44–47]. Considering that adjustment may not be sufficient to eliminate the imbalance between offspring born after fresh and frozen embryo transfer, we conducted a subgroup analysis. Our results showed that the long-term metabolic profiles were comparable between frozen embryos and fresh embryos, whether they were at the blastocyst or cleavage stage. However, it is important to note that the imbalance in numbers may have affected the interpretation of these results.

Thirdly, not all offspring provided blood samples in the whole “ARTKID” cohort. Possible reasons include failure to reach the follow-up deadline, offspring noncompliance with invasive blood testing, and external factors such as the COVID-19 pandemic during 2020 to 2021, or residing far from our center. We compared baseline characteristics between the included and not included population. The actual differences were relatively small. Thus, we would deduce that selection bias may have little impact on our results.

Fourthly, some potentially confounding information may not have been collected in our study, such as breastfeeding status and smoking during pregnancy. Breastfeeding may be associated with obesity in childhood and thereby the risk of metabolic syndrome [48]. However, there is no previous research focusing on the difference of breastfeeding between FET and Fresh-ET. Thus, it is difficult to assess the impact of breastfeeding on our results. It is well established that maternal tobacco exposure during pregnancy increases offspring obesity and metabolic risk [49,50]. But, only 3.4% of Chinese women were reported to be smokers in the 2010 national survey [51], and about 82.9% of smoking women quit smoking after becoming pregnant [52]. Therefore, the proportion of women smoking during pregnancy is extremely low in China. Moreover, maternal smoking may be unrelated to the selection of embryo transfers. Thus, this bias may have little impact on the results. Fifthly, genetic and socioeconomic factors that affect offspring could be additional potential confounders. A matched sibling cohort can help eliminate the influence of genetic factors, but it may significantly reduce the sample size and make it challenging to maintain offspring age balance. In our study, we collected and adjusted comprehensive parental baseline information to minimize its impact on the results as much as possible.

Another limitation is the “live-birth bias” from only focusing on live births in our cohorts [53]. However, live-birth is one essential inclusion criteria for assessing the long-term outcome of children, potentially inevitably introducing selection bias. Our study may only represent live birth offspring born from ART. Finally, the offspring were aged 2 to 5 years at the time of our study. As they are too young to reveal more comprehensive metabolic phenotypic changes, further longer follow-up studies may be needed.

In conclusion, compared to offspring born after fresh embryo transfer, those born after FET showed no significant adverse metabolic changes during early childhood in our large sample size study. However, the long-term metabolic health of offspring conceived through FET remains a concern, and continuous monitoring of their long-term metabolic health is necessary.

## Supporting information

S1 Table STROBE Statement—Checklist of items that should be included in reports of cohort studies. (DOCX)

S2 Table Pearson’s correlation coefficient of 7 outcome variables. (DOCX)

S3 Table Characteristics of included and nonincluded singleton children conceived from ART. (DOCX)

S4 Table Differential *p-*values and FDR for metabolic variables between offspring conceived by fresh versus frozen embryo transfer. (DOCX)

S5 Table Differential *p-*values and FDR for metabolic variables between offspring conceived by fresh versus frozen embryo transfer stratified by offspring sex. (DOCX)

S6 Table Differential *p-*values and FDR for metabolic variables between offspring conceived by fresh versus frozen embryo transfer stratified by stage of embryo transfer. (DOCX)

S7 Table Differential *p-*values and FDR for metabolic variables between offspring conceived by fresh versus frozen embryo transfer stratified by fertilization mode. (DOCX)

S8 Table Differences in metabolic variables between offspring conceived by fresh versus frozen embryo transfer after matching for offspring age. (DOCX)

S9 Table Differential *p-*values and FDR for metabolic variables between offspring conceived by fresh versus frozen embryo transfer after matching for offspring age. (DOCX)

S1 File The informed consent form. (DOCX)

S2 File The relevant policies of the Chinese government regarding follow-up. (DOCX)

S3 File Ethical approval document. (DOCX)

## Abbreviations

APOE: Apolipoprotein E
ART: assisted reproductive technology
ARTKID: Assisted Reproductive Technology borned KIDs
BMI: body mass index
DOHaD: Developmental Origins of Health and Disease
FBG: fasting blood glucose
FDR: false discovery rate
FET: frozen embryo transfer
GDM: gestational diabetes mellitus
GIUT: gamete intrauterine transfer
HDL-C: high-density lipoprotein-cholesterol
ICSI: intracytoplasmic sperm injection
IVF: in vitro fertilization
IVM: in vitro maturation
LDL-C: low-density lipoprotein-cholesterol
LGA: large for gestational age
MD: mean difference
OHSS: ovarian hyperstimulation syndrome
PGT: preimplantation genetic testing
PIH: pregnancy-induced hypertension
SDS: standard deviation score
TC: total cholesterol
TG: triglyceride
