# Supplementary material for: Metabolic profiles of children aged 2–5 years born after frozen and fresh embryo transfer: A Chinese cohort study
Source: PLoS Med. 2024 Jun 6;21(6):e1004388. doi: 10.1371/journal.pmed.1004388 (PMC11156393; doi:10.1371/journal.pmed.1004388)
Supplement: S2 Table — (DOCX) [file pmed.1004388.s002.docx]

**S2 Table.** Pearson’s correlation coefficient of 7 outcome variables.

|  | FBG | Insulin | HOMA-IR2 | TC | LDL-C | HDL-C | TG |
| --- | --- | --- | --- | --- | --- | --- | --- |
| FBG |  | **0.48** | **0.50** | 0.03 | 0.01 | -0.001 | 0.18 |
| Insulin | - |  | **0.99** | -0.02 | -0.02 | -0.06 | 0.24 |
| HOMA-IR2 | - | - |  | -0.02 | -0.02 | -0.06 | 0.24 |
| TC | - | - | - |  | **0.92** | **0.42** | 0.12 |
| LDL-C | - | - | - | - |  | 0.11 | 0.16 |
| HDL-C | - | - | - | - | - |  | -0.30 |
| TG | - | - | - | - | - | - |  |

Abbreviations: FBG, fasting blood glucose; HDL-C, high-density lipoprotein cholesterol; HOMA-IR2, homeostatic model assessment for insulin resistance using the HOMA2 Calculator; LDL-C, low-density lipoprotein cholesterol; TC, total cholesterol; TG, triacylglycerol.
