# Supplementary material for: Metabolic profiles of children aged 2–5 years born after frozen and fresh embryo transfer: A Chinese cohort study
Source: PLoS Med. 2024 Jun 6;21(6):e1004388. doi: 10.1371/journal.pmed.1004388 (PMC11156393; doi:10.1371/journal.pmed.1004388)
Supplement: S3 Table — (DOCX) [file pmed.1004388.s003.docx]

**S3 Table.** Characteristics of included and unincluded singleton children conceived from ART.

|  | **Fresh embryo transfer** | | | |  | **Frozen embryo transfer** | | | |
| --- | --- | --- | --- | --- | --- | --- | --- | --- | --- |
|  | **Unincluded (n=4309)** | **Included (n=2065)** | **MD or OR (95%CI)** | **P-value** |  | **Unincluded (n=5767)** | **Included (2181)** | **MD or OR (95%CI)** | **P-value** |
| **Parental characteristics** | |  |  |  |  |  |  |  |  |
| Maternal age, years | 31.8(28.8, 35.2) | 31.3(28.3, 34.8) | -0.36(-0.58, -0.14) ^#^ | 0.02 |  | 31.2(28.7, 34.5) | 30.8(28.3, 34) | -0.42(-0.62, -0.21) ^#^ | <0.001 |
| Paternal age, years **^†^** | 32.3(29.1, 36) | 31.9(28.8, 35.6) | -0.42(-0.67, -0.16) ^#^ | 0.001 |  | 31.9(29.1, 35.5) | 31.3(28.7, 34.8) | -0.52(-0.75, -0.28) ^#^ | <0.001 |
| Maternal BMI, kg/m^2^ **^†^** | 23(20.7, 25.7) | 22.8(20.8, 25.2) | -0.11(-0.30, 0.08) ^#^ | 0.26 |  | 22.7(20.6, 25.2) | 22.9(20.7, 25.4) | 0.17(-0.002, 0.35) ^#^ | 0.05 |
| Paternal BMI, kg/m^2^ **^†^** | 25.8(23.1, 28.4) | 25.5(23, 28.1) | -0.24(-0.45, -0.03) ^#^ | 0.27 |  | 25.6(23, 28.3) | 25.6(22.9, 28.1) | -0.03(-0.23, 0.17) ^#^ | 0.33 |
| Maternal diabetes, n (%) **^†^** | 24(0.6) | 6(0.3) | 0.52(0.21, 1.28) ^§^ | 0.15 |  | 18(0.3) | 5(0.2) | 0.73(0.27, 1.96) ^§^ | 0.53 |
| Paternal diabetes, n (%) **^†^** | 15(0.3) | 9(0.4) | 1.25(0.55, 2.87) ^§^ | 0.59 |  | 14(0.2) | 9(0.4) | 1.7(0.74, 3.94) ^§^ | 0.21 |
| Paternal smoking, n (%) | 1430(33.2) | 672(32.6) | 0.97(0.87, 1.09) ^§^ | 0.62 |  | 1933(33.5) | 703(32.3) | 0.95(0.85, 1.05) ^§^ | 0.29 |
| Parity (0), n (%) | 3493(81.1) | 1725(83.5) | 1.19(1.03, 1.36) ^§^ | 0.02 |  | 4573(79.3) | 1796(82.3) | 1.22(1.07, 1.38) ^§^ | 0.002 |
| Maternal education (college or higher), n (%) | 1506(35) | 662(32.1) | 0.88(0.79, 0.98) ^§^ | 0.02 |  | 1990(34.5) | 706(32.4) | 0.91(0.82, 1.01) ^§^ | 0.07 |
| Paternal education (college or higher), n (%) | 1646(38.2) | 773(37.4) | 0.97(0.87, 1.08) ^§^ | 0.56 |  | 2226(38.6) | 790(36.2) | 0.9(0.82, 1.001) ^§^ | 0.05 |
| **ART characteristics** | |  |  |  |  |  |  |  |  |
| Blastocyst embryo transfer, n (%) | 814(18.9) | 371(18) | 0.94(0.82, 1.08) ^§^ | 0.38 |  | 5593(97) | 2052(94.1) | 0.5(0.39, 0.63) ^§^ | <0.001 |
| ICSI used, n (%) | 1186(27.5) | 655(31.7) | 1.22(1.09, 1.37) ^§^ | 0.001 |  | 1372(23.8) | 609(27.9) | 1.24(1.11, 1.39) ^§^ | <0.001 |
| **Pregnancy and offspring characteristics** | | |  |  |  |  |  |  |  |
| GDM, n (%) | 324(7.5) | 147(7.1) | 0.94(0.77, 1.15) ^§^ | 0.57 |  | 176(8.1) | 428(7.4) | 1.1(0.91, 1.32) ^§^ | 0.33 |
| PIH, n (%) | 182(4.2) | 84(4.1) | 0.96(0.74, 1.25) ^§^ | 0.77 |  | 374(6.5) | 141(6.5) | 1(0.82, 1.22) ^§^ | 0.97 |
| Offspring sex (male), n (%) | 2215(51.4) | 1080(52.3) | 1.04(0.93, 1.15) ^§^ | 0.50 |  | 3042(52.7) | 1190(54.6) | 1.08(0.97, 1.19) ^§^ | 0.15 |
| Cesarean section, n (%) | 2873(66.7) | 1486(72) | 1.28(1.14, 1.44) ^§^ | <0.001 |  | 4078(70.7) | 1634(74.9) | 1.24(1.11, 1.38) ^§^ | <0.001 |
| Gestational age, weeks | 278(6.5) | 141(6.8) | 1.06(0.86, 1.31) ^#^ | <0.001 |  | 382(6.6) | 141(6.5) | 0.97(0.8, 1.19) ^#^ | 0.02 |
| Birth weight, kg | 3.4(3.1, 3.7) | 3.4(3.1, 3.7) | -0.01(-0.04, 0.02) ^#^ | 0.57 |  | 3.5(3.15, 3.8) | 3.5(3.2, 3.8) | 0.03(-0.001, 0.05) ^#^ | 0.06 |

Data were presented as interquartile range (25^th^ percentile, 75^th^ percentile) or n (%).

Mean differences or odds ratio were obtained using a regression model.

^#^ For continuous variables, the regression results are presented as mean difference (95% CI).

^§^ For categorical variables, the regression results are presented as odds ratios (95% CI).

**^†^** Sixty-one data were missing in the parental BMI, 67 data were missing in the paternal age, and 176 data were missing in the parental diabetes.

Abbreviations: ART, assisted reproductive technology; BMI, body mass index; GDM, Gestational Diabetes Mellitus; ICSI, intracytoplasmic sperm injection; IVF, in vitro fertilization; MD, mean difference; OR, odds ratio; PIH, pregnancy-induced hypertension syndrome.
