# Supplementary material for: Metabolic profiles of children aged 2–5 years born after frozen and fresh embryo transfer: A Chinese cohort study
Source: PLoS Med. 2024 Jun 6;21(6):e1004388. doi: 10.1371/journal.pmed.1004388 (PMC11156393; doi:10.1371/journal.pmed.1004388)
Supplement: S5 Table — (DOCX) [file pmed.1004388.s005.docx]

**S5 Table.** Differential p-values and FDR for metabolic variables between offspring conceived by fresh versus frozen embryo transfer stratified by offspring sex.

|  | **Crude Model** | |  | **Adjusted Model** | |  |
| --- | --- | --- | --- | --- | --- | --- |
|  | ***P* value** | **FDR q value** |  | ***P* value** | **FDR q value** |  |
| **Female Offspring** |  |  |  |  |  |  |
| FBG | 0.27 | 0.54 |  | 0.54 | 0.88 |  |
| Insulin | 0.77 | - |  | 0.63 | - |  |
| HOMA-IR2 | 0.78 | - |  | 0.64 | - |  |
| TC | 0.51 | - |  | 0.64 | - |  |
| TG | 0.62 | 0.80 |  | 0.66 | 0.88 |  |
| LDL-C | 0.19 | 0.54 |  | 0.23 | 0.61 |  |
| HDL-C | 0.23 | 0.54 |  | 0.14 | 0.56 |  |
| **Male Offspring** |  |  |  |  |  |  |
| FBG | 0.42 | 0.67 |  | 0.86 | 0.95 |  |
| Insulin | 0.40 | - |  | 0.93 | - |  |
| HOMA-IR2 | 0.38 | - |  | 0.95 | - |  |
| TC | 0.79 | - |  | 0.83 | - |  |
| TG | 0.80 | 0.80 |  | 0.95 | 0.95 |  |
| LDL-C | 0.74 | 0.80 |  | 0.66 | 0.88 |  |
| HDL-C | 0.14 | 0.54 |  | 0.09 | 0.56 |  |

FDR were obtained using Benjamin-Hochberg's procedure.

Adjusted Model: adjusted for maternal age, paternal age, maternal BMI, paternal BMI, maternal education, paternal education, paternal smoking, parity and offspring age.

Abbreviations: FBG, fasting blood glucose; FDR, false discovery rate; HDL-C, high-density lipoprotein cholesterol; HOMA-IR2, homeostatic model assessment for insulin resistance using the HOMA2 Calculator; LDL-C, low-density lipoprotein cholesterol; TC, total cholesterol; TG, triacylglycerol.
