# Supplementary material for: Metabolic profiles of children aged 2–5 years born after frozen and fresh embryo transfer: A Chinese cohort study
Source: PLoS Med. 2024 Jun 6;21(6):e1004388. doi: 10.1371/journal.pmed.1004388 (PMC11156393; doi:10.1371/journal.pmed.1004388)
Supplement: S6 Table — (DOCX) [file pmed.1004388.s006.docx]

**S6 Table.** Differential *p*-values and FDR for metabolic variables between offspring conceived by fresh versus frozen embryo transfer by stage of embryo transfer.

|  | **Crude Model** | |  | **Adjusted Model** | |
| --- | --- | --- | --- | --- | --- |
|  | ***P* value** | **FDR q value** |  | ***P* value** | **FDR q value** |
| **Cleavage** |  |  |  |  |  |
| FBG | 0.90 | 0.90 |  | 0.68 | 0.73 |
| Insulin | 0.16 | - |  | 0.35 | - |
| HOMA-IR2 | 0.21 | - |  | 0.40 | - |
| TC | 0.15 | - |  | 0.18 | - |
| TG | 0.14 | 0.53 |  | 0.20 | 0.59 |
| LDL-C | 0.45 | 0.6 |  | 0.49 | 0.65 |
| HDL-C | 0.33 | 0.53 |  | 0.35 | 0.59 |
| **Blastocyst** |  |  |  |  |  |
| FBG | 0.27 | 0.53 |  | 0.24 | 0.59 |
| Insulin | 0.18 | - |  | 0.25 | - |
| HOMA-IR2 | 0.19 | - |  | 0.26 | - |
| TC | 0.48 | - |  | 0.55 | - |
| TG | 0.004 | 0.03 |  | 0.01 | 0.08 |
| LDL-C | 0.62 | 0.71 |  | 0.73 | 0.73 |
| HDL-C | 0.29 | 0.53 |  | 0.37 | 0.59 |

FDR were obtained using Benjamin-Hochberg's procedure.

Adjusted Model: adjusted for maternal age, paternal age, maternal BMI, paternal BMI, maternal education, paternal education, paternal smoking, parity, offspring age and sex.

Abbreviations: FBG, fasting blood glucose; FDR, false discovery rate; HDL-C, high-density lipoprotein cholesterol; HOMA-IR2, homeostatic model assessment for insulin resistance using the HOMA2 Calculator; LDL-C, low-density lipoprotein cholesterol; TC, total cholesterol; TG, triacylglycerol.
