# Supplementary material for: Metabolic profiles of children aged 2–5 years born after frozen and fresh embryo transfer: A Chinese cohort study
Source: PLoS Med. 2024 Jun 6;21(6):e1004388. doi: 10.1371/journal.pmed.1004388 (PMC11156393; doi:10.1371/journal.pmed.1004388)
Supplement: S8 Table — (DOCX) [file pmed.1004388.s008.docx]

**S8 Table.** Differences in metabolic variables between offspring conceived by fresh versus frozen embryo transfer after matching for offspring age.

|  | **Fresh embryo transfer**  **(n=1946)**  **(2114 visits)** | **Frozen embryo transfer**  **(n=1966)**  **(2114 visits)** | **Crude Model MD**  **(95%CI)** | ***P* value** | **Adjusted Model MD**  **(95%CI)** | ***P* value** |
| --- | --- | --- | --- | --- | --- | --- |
| FBG **^†^**, mmol/l | 4.89±0.45 | 4.88±0.42 | -0.01(-0.03, 0.02) | 0.70 | -0.004(-0.03, 0.02) | 0.75 |
| Insulin **^†^**, mIU/l | 4.20±3.04 | 4.18±2.91 | -0.03(-0.21, 0.15) | 0.74 | 0(-0.18, 0.18) | 0.99 |
| HOMA-IR2 **^†^** | 0.55±0.40 | 0.55±0.38 | -0.004(-0.03, 0.02) | 0.72 | 0(-0.02, 0.02) | 0.97 |
| TC **^†^**, mmol/l | 4.07±0.73 | 4.07±0.72 | -0.01(-0.06, 0.03) | 0.61 | -0.01(-0.05, 0.04) | 0.77 |
| TG **^†^**, mmol/l | 0.73±0.29 | 0.73±0.30 | 0(-0.18, 0.18) | 0.99 | 0(-0.02, 0.02) | 0.93 |
| LDL-C **^†^**, mmol/l | 2.45±0.61 | 2.43±0.60 | -0.03(-0.06, 0.01) | 0.17 | -0.02(-0.06, 0.02) | 0.25 |
| HDL-C **^†^**, mmol/l | 1.39±0.29 | 1.41±0.30 | 0.02(-0.0003, 0.04) | 0.05 | 0.02(0.001, 0.04) | 0.04 |

Note: The offspring age at visit following matching were 3.59±0.72 and 3.58±0.76, respectively. And the *p*-value resulting from the *t*-test was 0.45.

Data were presented as mean ± SD.

Mean differences were obtained using a linear mixed-effects regression model.

Adjusted Model: adjusted for maternal age, paternal age, maternal BMI, paternal BMI, maternal education, paternal education, paternal smoking, parity, offspring age and sex.

**^†^** Twenty data were missing in the FBG, 5 data were missing in the insulin, 25 data were missing in the HOMA-IR2, and 19 data were missing in the lipid metabolic variables.

Abbreviations: FBG, fasting blood glucose; HDL-C, high-density lipoprotein cholesterol; HOMA-IR2, homeostatic model assessment for insulin resistance using the HOMA2 Calculator; LDL-C, low-density lipoprotein cholesterol; MD, mean difference; TC, total cholesterol; TG, triacylglycerol.
