# Supplementary material for: Metabolic profiles of children aged 2–5 years born after frozen and fresh embryo transfer: A Chinese cohort study
Source: PLoS Med. 2024 Jun 6;21(6):e1004388. doi: 10.1371/journal.pmed.1004388 (PMC11156393; doi:10.1371/journal.pmed.1004388)
Supplement: S9 Table — (DOCX) [file pmed.1004388.s009.docx]

**S9 Table.** Differential *p*-values and FDR for metabolic variables between offspring conceived by fresh versus frozen embryo transfer after matching for offspring age.

|  | **Crude Model** | |  | **Adjusted Model** | |
| --- | --- | --- | --- | --- | --- |
|  | ***P* value** | **FDR q value** |  | ***P* value** | **FDR q value** |
| FBG | 0.70 | 0.93 |  | 0.75 | 0.93 |
| Insulin | 0.74 | - |  | 0.99 | - |
| HOMA-IR2 | 0.72 | - |  | 0.97 | - |
| TC | 0.61 | - |  | 0.77 | - |
| TG | 0.99 | 0.99 |  | 0.93 | 0.93 |
| LDL-C | 0.17 | 0.34 |  | 0.25 | 0.50 |
| HDL-C | 0.05 | 0.20 |  | 0.04 | 0.16 |

FDR were obtained using Benjamin-Hochberg's procedure.

Adjusted Model: adjusted for maternal age, paternal age, maternal BMI, paternal BMI, maternal education, paternal education, paternal smoking, parity, offspring age and sex.

Abbreviations: FBG, fasting blood glucose; FDR, false discovery rate; HDL-C, high-density lipoprotein cholesterol; HOMA-IR2, homeostatic model assessment for insulin resistance using the HOMA2 Calculator; LDL-C, low-density lipoprotein cholesterol; TC, total cholesterol; TG, triacylglycerol.
