# Supplementary material for: Metabolic profiles of children aged 2–5 years born after frozen and fresh embryo transfer: A Chinese cohort study
Source: PLoS Med. 2024 Jun 6;21(6):e1004388. doi: 10.1371/journal.pmed.1004388 (PMC11156393; doi:10.1371/journal.pmed.1004388)
Supplement: S1 File — (DOCX) [file pmed.1004388.s010.docx]

**S1 File.** The informed consent form.

The informed consent form was shown below. Participants voluntarily agree to participate in the follow-up assessments for themselves and their offspring upon signing the informed consent.

**Highlight 1:** According to the Ministry of Health's "Ethical Principles of Human Assisted Reproductive Technology and Human Sperm Bank" and "Regulations on Human Assisted Reproductive Technology," it is stipulated that the follow-up rate for human assisted reproductive technology should be above 95%. Infertile couples receiving assisted reproductive technology services are obliged to cooperate with medical institutions to complete follow-up procedures.

***
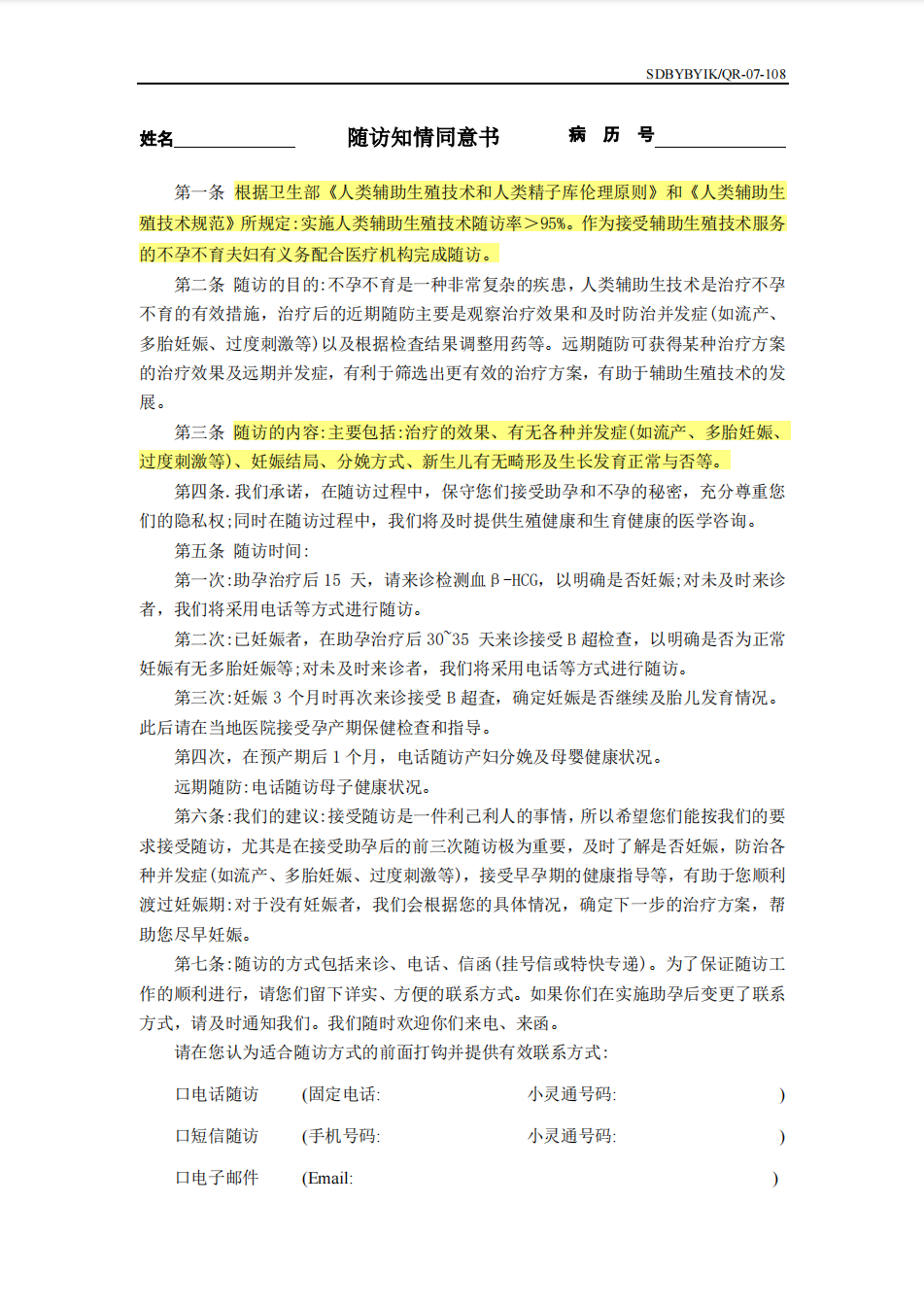
*Highlight 2:** The content of the follow-up mainly includes: effect of treatment, presence of various complications (such as miscarriage, multiple pregnancies, ovarian hyperstimulation syndrome, etc.), pregnancy outcomes, mode of delivery, presence of newborn abnormalities, and whether growth and development are normal.


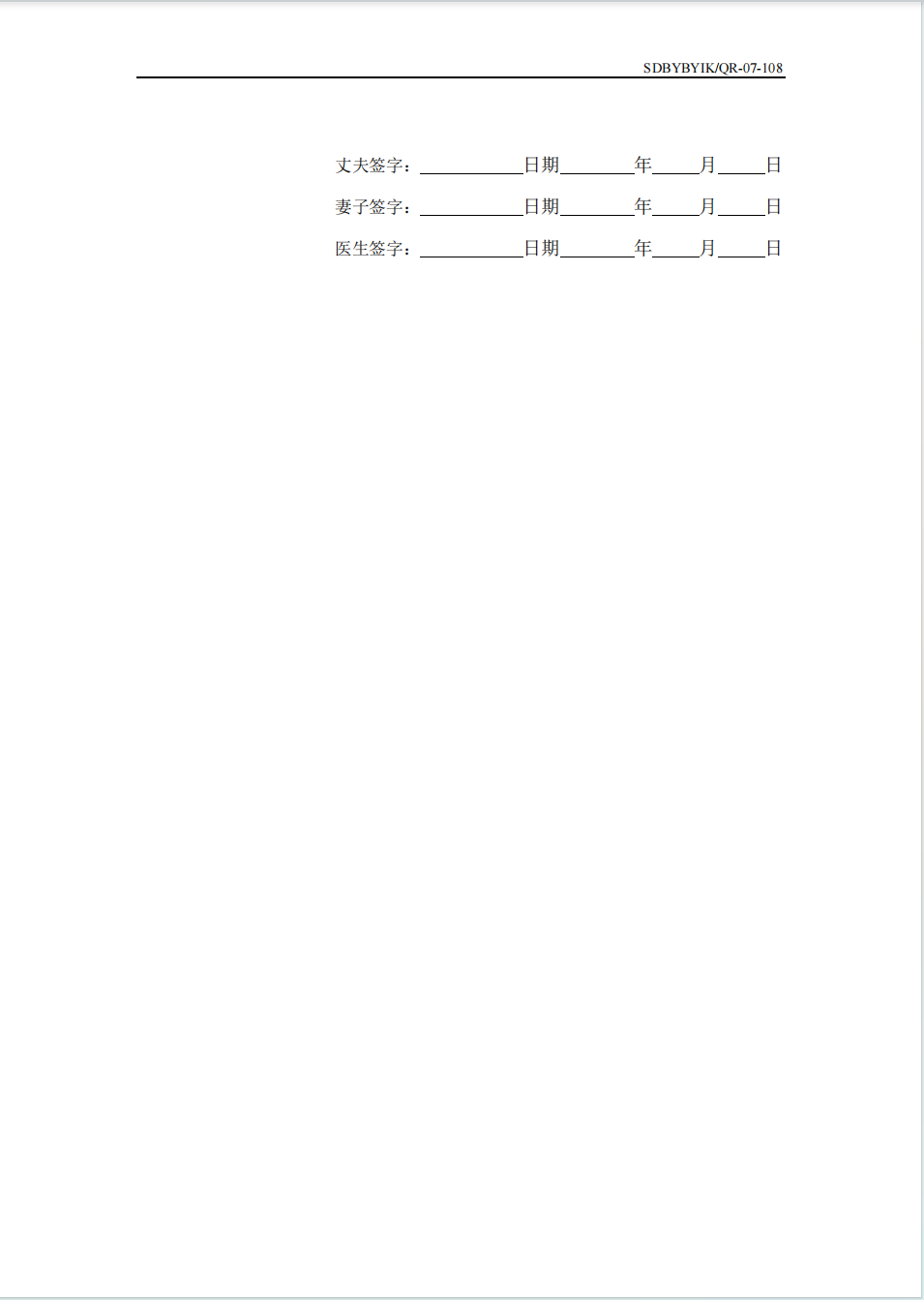


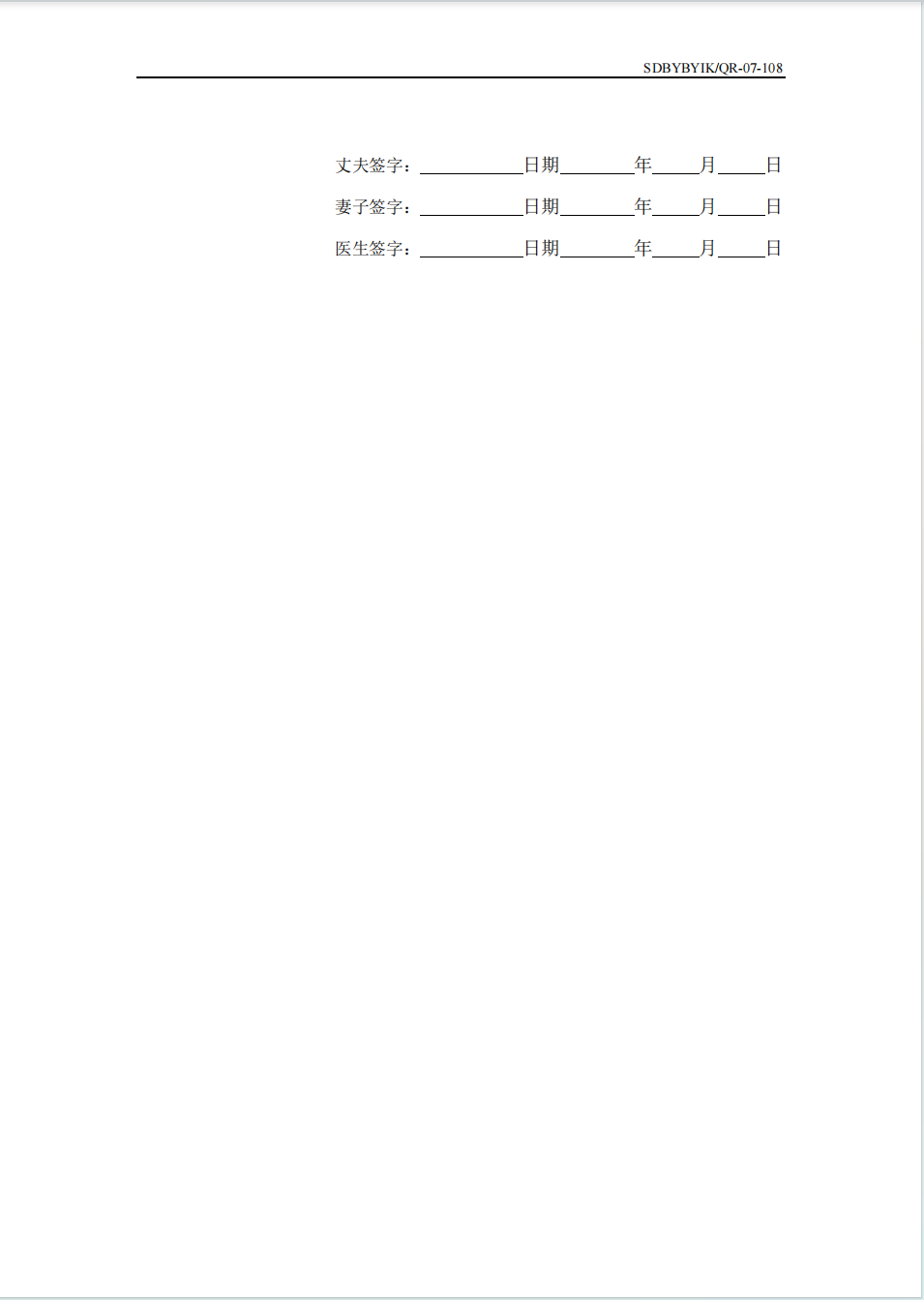


The informed consent form in English. (Translated by the authors of this paper)

**
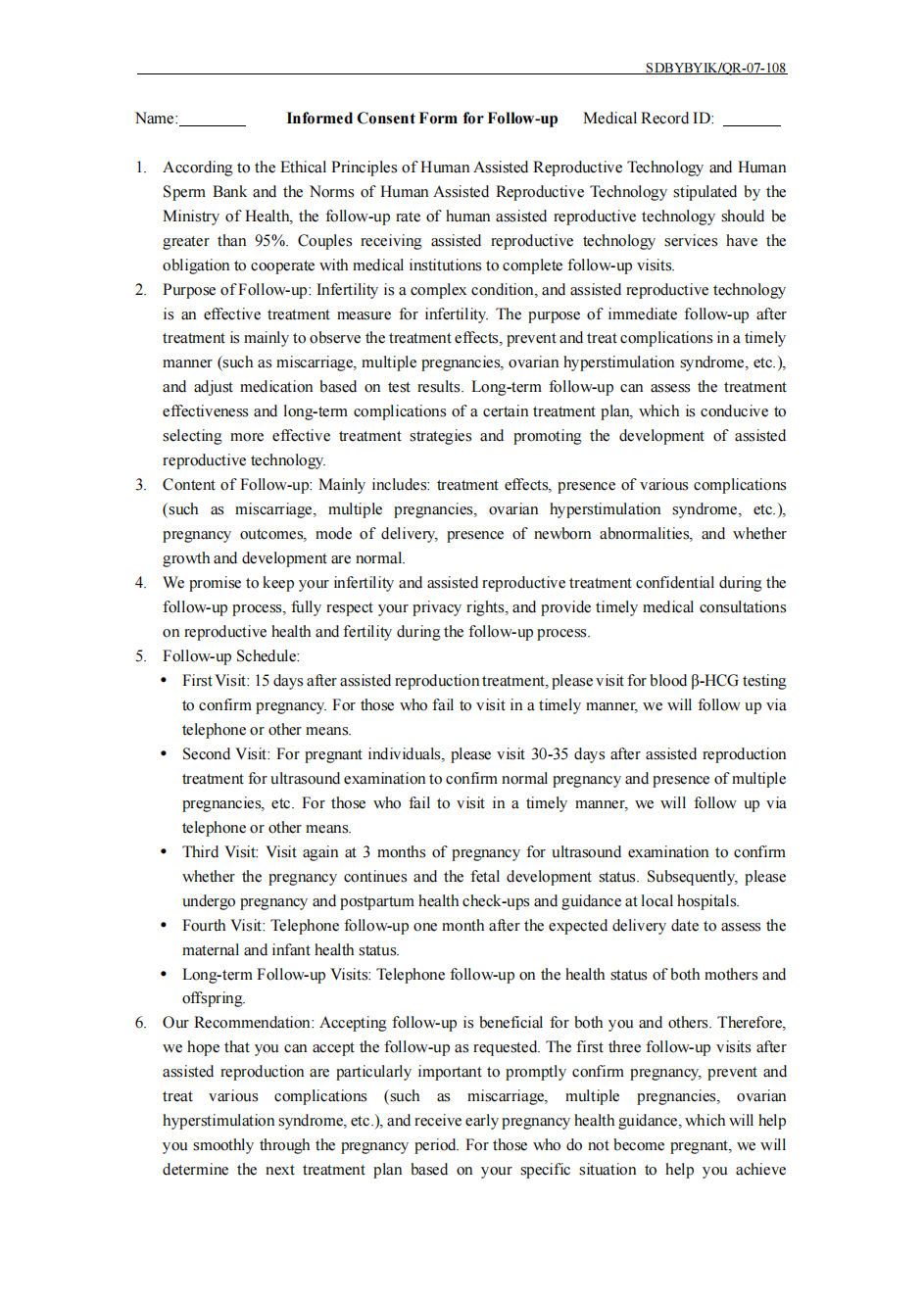
**

**
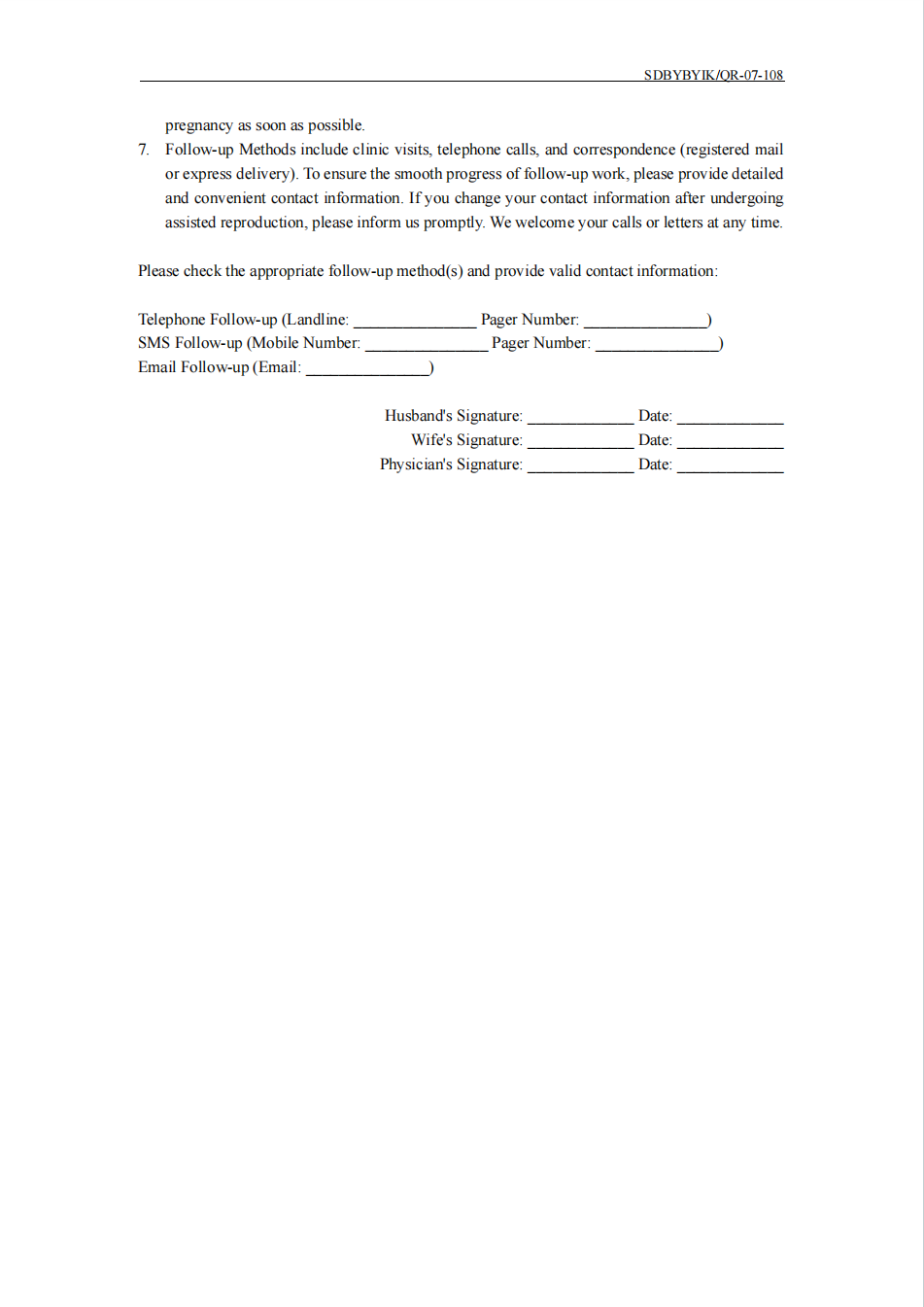
**
