# Supplementary material for: Metabolic profiles of children aged 2–5 years born after frozen and fresh embryo transfer: A Chinese cohort study
Source: PLoS Med. 2024 Jun 6;21(6):e1004388. doi: 10.1371/journal.pmed.1004388 (PMC11156393; doi:10.1371/journal.pmed.1004388)
Supplement: S2 File — (DOCX) [file pmed.1004388.s011.docx]

**S2 File.** The relevant policies of the Chinese government regarding follow-up.

In the 2003 document issued by the Chinese Ministry of Health, it is stipulated that assisted reproductive institutions should establish a follow-up system. (Details: <http://www.nhc.gov.cn/wjw/gfxwj/200309/b2c02b9bf1fc427586fc3905c5c2df87.shtml>)

In vitro fertilization-embryo transfer and related technology standards (Translated by the authors of this paper)

Basic requirements:

6. Regulations

Institutions should establish the following systems:

(1) Reproductive Medicine Ethics Committee working system;

(2) Medical records management system;

(3) Follow-up system;

(4) Staff division of labor and responsibility system;

(5) Quality control system for handling experimental materials of gametes and embryos;

(6) Routine technical operation procedures;

(7) Management system for special drugs;

(8) Instrument management system;

(9) Disinfection and isolation system;

(10) Material management system.
