# Supplementary material for: Metabolic profiles of children aged 2–5 years born after frozen and fresh embryo transfer: A Chinese cohort study
Source: PLoS Med. 2024 Jun 6;21(6):e1004388. doi: 10.1371/journal.pmed.1004388 (PMC11156393; doi:10.1371/journal.pmed.1004388)

**S3 File.** Ethical approval document.

Title of the profile: Research project ethical approval document

Title of the study: The Effects of Assisted Reproductive Technology on Maternal and Offspring Health: ART Cohort Study

Ethical approval number: [2014] (17).


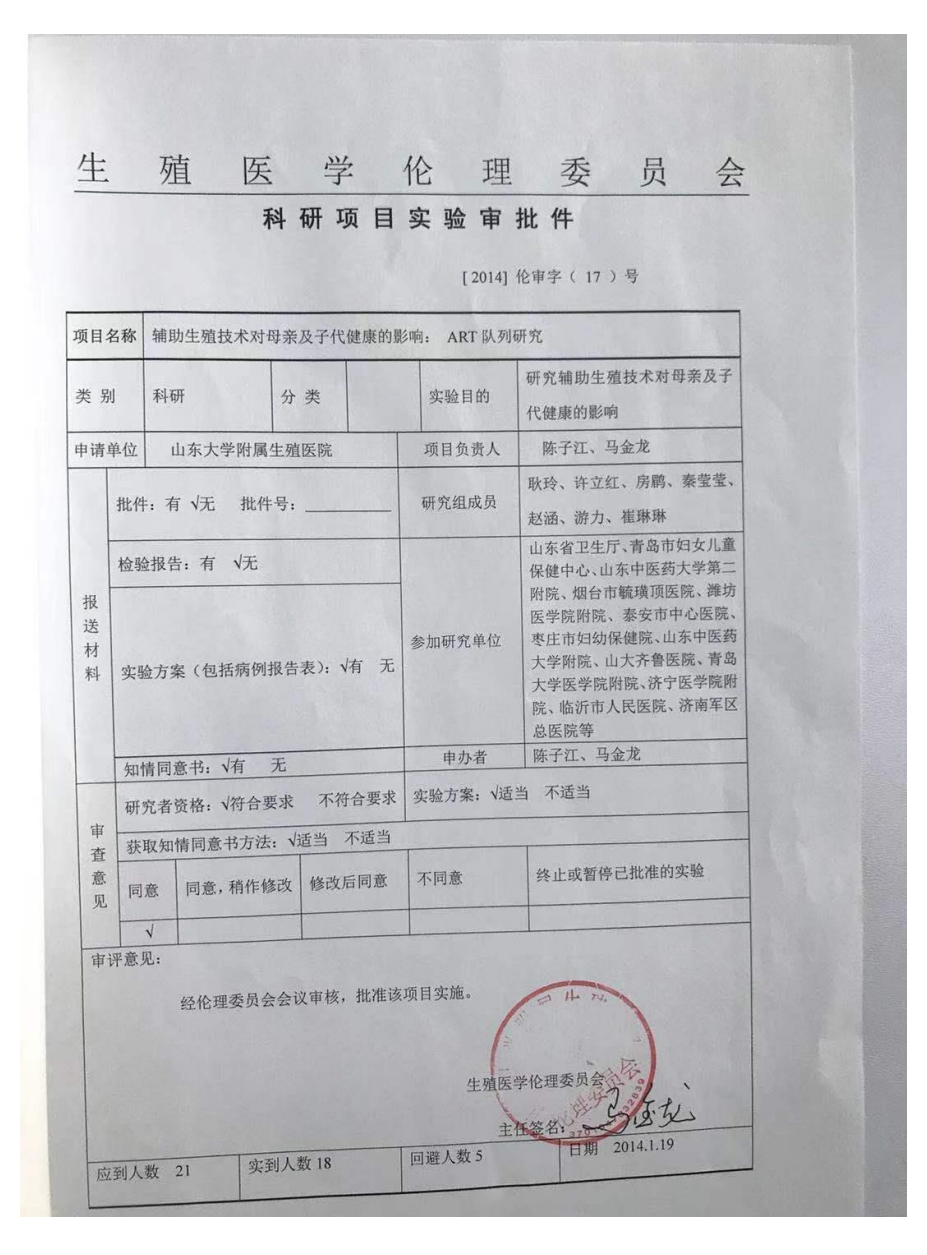
Ethical approval document in English. (Translated by the authors of this paper)
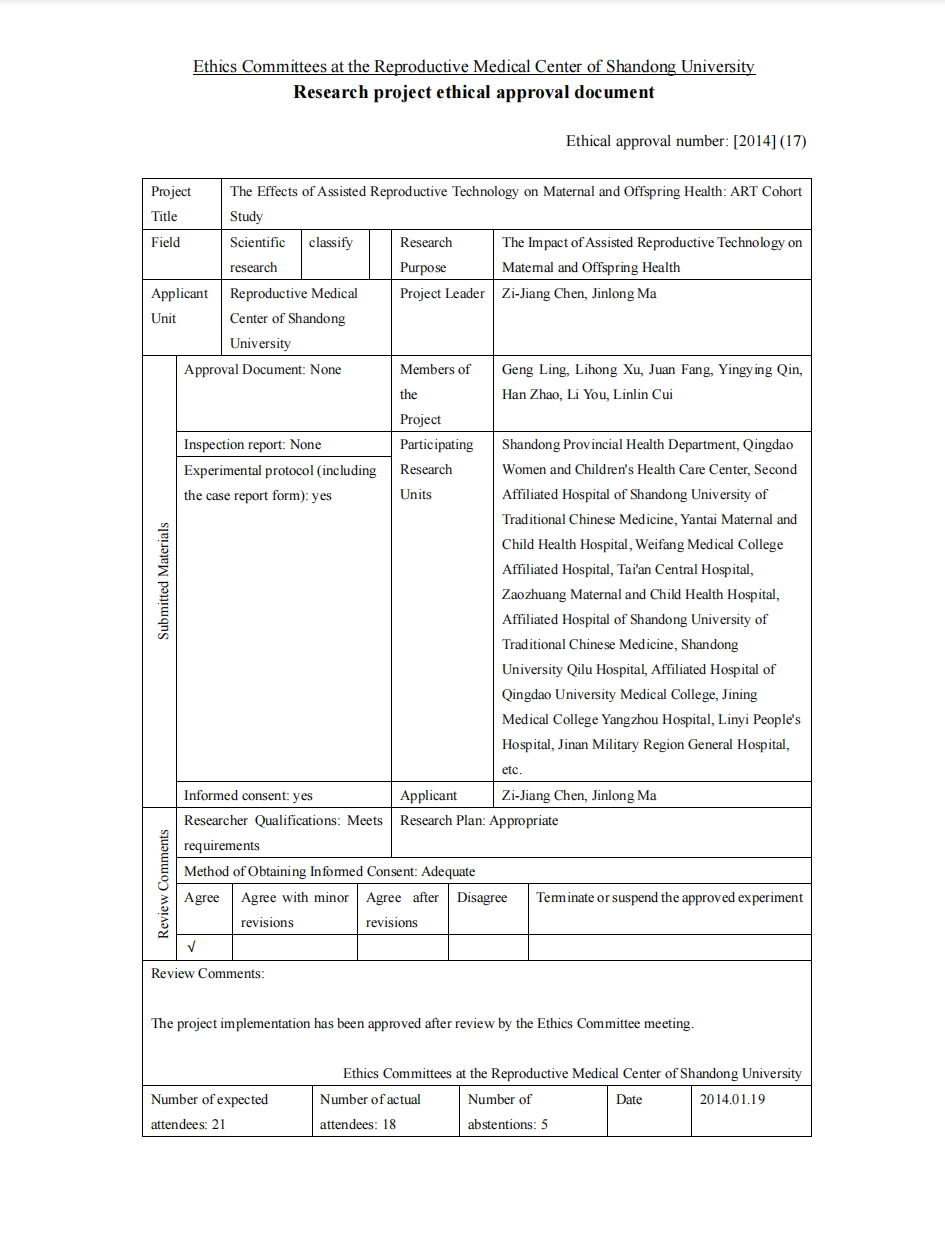

Supplement: S3 File — (DOCX) [file pmed.1004388.s012.docx]
